# The Potential of Grape Pomace Varieties as a Dietary Source of Pectic Substances

**DOI:** 10.3390/foods10040867

**Published:** 2021-04-15

**Authors:** Mariana Spinei, Mircea Oroian

**Affiliations:** Department of Food Technologies, Food Production and Environment Safety, Faculty of Food Engineering, Stefan cel Mare University of Suceava, 720229 Suceava, Romania; mariana.spinei@fia.usv.ro

**Keywords:** grape pomace, grape skin, grape seeds, grape inflorescence architectures, pectin, extraction

## Abstract

Grape pomace is one of the most abundant solid by-products generated during winemaking. A lot of products, such as ethanol, tartrates, citric acid, grape seed oil, hydrocolloids, bioactive compounds and dietary fiber are recovered from grape pomace. Grape pomace represents a major interest in the field of fiber extraction, especially pectin, as an alternative source to conventional ones, such as apple pomace and citrus peels, from which pectin is obtained by acid extraction and precipitation using alcohols. Understanding the structural and functional components of grape pomace will significantly aid in developing efficient extraction of pectin from unconventional sources. In recent years, natural biodegradable polymers, like pectin has invoked a big interest due to versatile properties and diverse applications in food industry and other fields. Thus, pectin extraction from grape pomace could afford a new reason for the decrease of environmental pollution and waste generation. This paper briefly describes the structure and composition of grape pomace of different varieties for the utilization of grape pomace as a source of pectin in food industry.

## 1. Introduction

The main by-products of the wine industry are grape pomace, grape seeds, grape bunches, yeast and tartrates sediments [1]. Grape pomace represents the residue from pressing process of fresh grapes, which are fermented or not. Grape pomace is one of the most important residue obtained in wine industry and constitutes 20–25% of grape weight [2] which contains skin, seeds and other solid parts. This by-product represents a complex substrate composed of 30% neutral polysaccharides, 20% pectic substances, 15% insoluble proanthocyanidins, structural proteins and phenolic compounds [3,4,5,6,7].

Pectic substances are a class of complex polysaccharide found in the cell walls of higher plants which act like as moisturizing agent and resistance material for cellulose network [8]. The majority of plants contain pectin in the intercellular layer between primary cell wall of adjacent cells [9]. Pectin are a complex group of polysaccharides which consisted of a chain of galacturonic acid units which are linked by α-1,4 glycosidic bonds [10].

Nowadays, there are several unconventional sources of pectin which have different physico-chemical properties, but there was not any research based on the studying structure and composition of grape pomace from the perspective of pectin source. Thus, in the context of another unconventional source of pectin, we proposed to investigate the composition of grape pomace (skin, seeds and inflorescence architectures) and characterized the grape pomace pectin. In addition, we presented the actual state of studies in the field of soluble fiber extraction from grape pomace and the potential of different grape varieties for pectin production.

## 2. Structure and Composition of Grape Pomace

**Figure 1:**
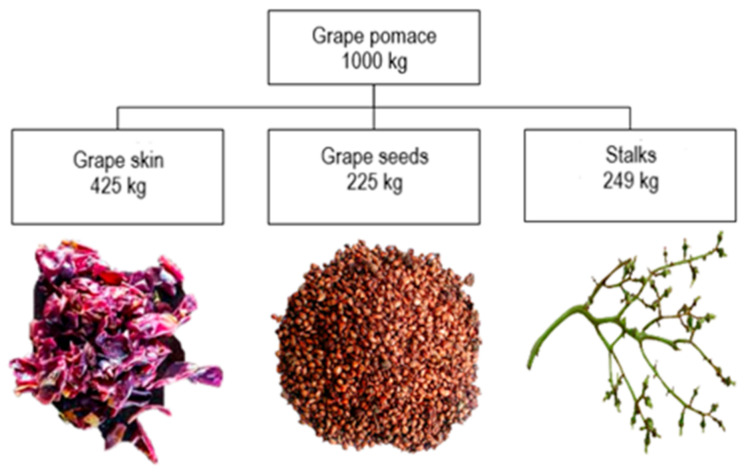
Composition of grape pomace reported to 1000 kg [1].

Grape pomace is the result of pressing whole grapes during the production of must. The amount of grape pomace represents around 20–25% of the mass of total processed grapes [11,12,13,14] and depends on the *terroir*, grape variety, degree of grape ripeness and the type of press used in the production process [15,16,17]. According to assessments, 1 kg of grape pomace is generated for each 6 L of wine [2,18]. One tone of grape pomace is consisted of 425 kg of grape skin, 225 kg of grape seeds, 249 kg of stalks and other minor constituents (e.g., water) (Figure 1) [1].

Regarding the composition of grape pomace, the moisture content varies from 50% to 72%, being influenced by the variety and degree of grape ripeness. Lignin represents 16.8% to 24.2% of the pomace compounds, while the protein content is less than 4% of the total grape pomace compounds [19]. In general, pectic substances are the main polymeric components of the grape cell walls, with values of 37–54% of the total cell wall polysaccharides. Cellulose is the second type of cell wall polysaccharides (27–37%) [4]. A wide range of products, such as ethanol, tartrates, citric acid, oil, hydrocolloids, dietary fiber are recovered from grape pomace [20,21,22]. Grape pomace is abundant in polyphenols—resveratrol, anthocyanins, flavones and tannins [23,24,25,26,27,28,29,30,31,32]. The grape pomace can be considered an unconventional source of pectin [24,33,34,35,36]. Table 1 describes the physico-chemical composition of grape pomace (*Vitis vinifera* L.) in terms of physico-chemical parameters, mineral and bioactive compounds related to the dry matter content.

### 2.1. Structure and Composition of Grape Skin

**Figure 2:**
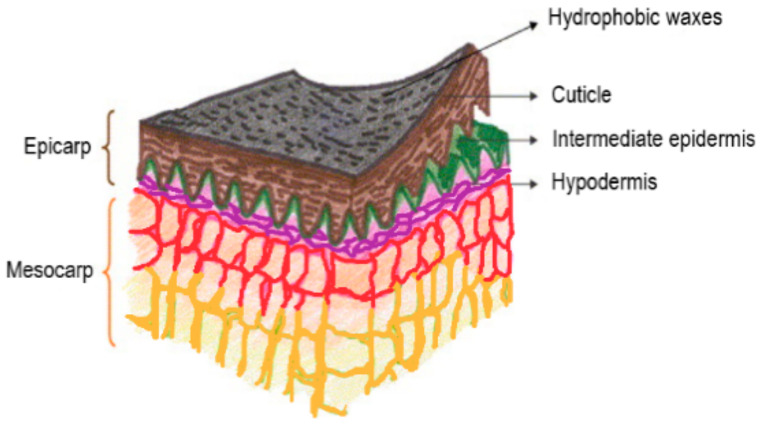
Different layers of the grape skin [3].

Grape skin represents about 5–10% of the total grape weight and serve as a hydrophobic barrier to protect the grapes from physical and climatic damages [3]. The grape skin could be separated into three overlapped stratums (Figure 2): (1) the outer stratum, the cuticle, is consisted of saturated and unsaturated carboxylic acids and coated by hydrophobic coatings; (2) the middle stratum (epidermis), is composed of one or two stratums, which represents a typical formation of cells; and (3) the interior stratum (subcutaneous tissue), is made up of different cell layers, which contains most of the phenolic compounds in grape skin [40]. The cell wall (CW) of grape berries establishes an obstruction to the diffusion of bioactive compounds (e.g., aromas, phenols and anthocyanins) and forms a barrier opposed to physical factors [41]. Grape skin CW consists of 30% benign polysaccharides (galactan, cellulose, xyloglucan, arabinan, xylan and mannan), 20% acidic pectic substances (63% are methyl esterified), ≈15% insoluble proanthocyanidins and less than 5% of structural proteins [3,18,42,43,44,45]. Three important tiers form the CW of grape berries [46]. (1) The middle lamella, which ties up the cells, is primarily made up of pectin. (2) The main CW, which is denser than the intermediate lamella and is built by the distribution of cells. The CW includes three structurally distinct, but connecting fractions: the two first fractions contain the essential cellulose (8–25%)–xyloglucan (25–50%) structure which is encapsulated in a pectin polysaccharides framework (10–35%). The third fraction includes structural proteins (10%) [46,47]. (3) The secondary CW is composed of cellulose microfibrils, arranged in parallel bunches (40–80%). The secondary CW also includes hemicelluloses (10–40%), pectins and lignin (5–25%) [47].

#### 2.1.1. Polysaccharide Structures in Grape Skin

##### The Cellulose–Xyloglucan Framework

Cellulose is consisted of D-glucose units which condense through β(1→4)-glycosidic bonds. Hydrogen bonding comprise 40 of cellulose chains to make microfibrils [48,49]. In CW of grape skin, hemicellulosic polysaccharides represent mostly of xyloglucans whose structures are established on a β(1→4) D-glucan backbone with approximately 75% of the glucose residues bring α(1→6) D-xylose residues, about 35% of which are replaced with fucosylated galactose residues [50]. Other hydrolysable monosaccharides like mannans (mannose–glucose backbone with galactose units attributed), xylans (xylose backbone with arabinose units attributed) and arabino-galactans (arabinose and galactose chains) were found in small amounts in grape skin CW [51]. In general, in flowering plants, xyloglucans are supposed to relate non-covalently with cellulose and establish two different parts assembling an interlaced chain: one attaches rigidly by hydrogen links to the uncovered regions of the glucan bonds in the cellulose nanoparticles and a following part which includes the length to the nanoparticle or interlink with other xyloglucans, as in a chain-link bond, to thin and clasp the nanoparticles inside region [38,52,53]. Park and Cosgrove [52] have showed the probability of covalent conjunctions between portions of xyloglucans into the CW structure. Moreover, cellulose and hemicellulose are more structurally organized in the secondary than in primary part of the CWs. Thus, Gao et al. [53] have reported that ether and ester bindings may associate hemicellulose to non-core lignin in the secondary part of CWs.

##### Pectin Polysaccharides

The major pectin polysaccharides identified in grape skin are homogalacturonan, consisting of a linear chain of α(1→4) *D*-galacturonic acid (GalA), rhamnogalacturonan I, containing a backbone of up 100 repeats of the dimer [→4)-α-*D*-Gal*p*A-(1→2)-α-*L*-Ram*p*-(1→] and rhamnogalacturonan II which includes α(1→4) *D*-Gal*p*A residues with 13 different monosaccharides [45,51,54,55,56,57]. At the present time, there are some research works based on physico-chemical techniques which explain the linkage of pectin to the cellulose-xyloglucan framework in the CWs of the grape skin, but some of them are contradictory. Fasoli et al. [58] have been deduced that a covalent interconnection can exist between xyloglucan chains and neutral sugar-rich pectic fragments, the latter are retained together partially by Ca^2+^ bridges [59]. Several authors established the formation of hemicellulose–pectin cross-linked networks in different plant CWs [60,61,62]. Pectins are encapsulated within cellulose/hemicellulose network, forming hydrophilic gels which determine mechanical characteristics of the CW, such as water holding capacity, regulation of ion transport and permeability of the wall for enzymes. The structure of pectin polysaccharides is influenced by its neutral sugar content, proportions of smooth and hairy regions, amounts of methoxyl and acetyl esters, molar mass and ferulic acid substitution.

##### Role of Ferulic Acid in Cross-Links among Sugars

The ferulic acid has an essential function in strengthening the structure of plant CWs by forming cross-links between polysaccharides and proteins, polysaccharides, polysaccharide chains and lignin [63,64]. Oligomerization of the feruloylated polysaccharides binds these CW components together with involvement for the physiological functions of the CW with respect to growth cessation, extensibility and improving recalcitrance on enzymatic degradation and microorganism activity [64,65]. Grabber et al. [66] have been reported the role of ferulic acid in the framework of graminaceous plants, like maize. Ester-linked ferulic acid bonds to α-arabinose backbone on xylans; thus, xylans are ester-coupled by ferulate’s peroxidase/H_2_O_2_-mediated radical linking into 8-8′, 8-5′, 8-O-4 and 5-5′-coupled dehydrodimers. Waldron et al. [67] studied the function of feruloylated structures in two closely related *Chenopodiaceae* species, sugar beet and beetroot and noted that in sugar beet 20% of the feruloyl components were incorporated into dimers in comparison to only 10% in beetroot.

##### Lignin

Lignin is not a carbohydrate because it is composed of different acids, such as *p*-coumaric, sinapic, cinnamic, ferulic, diferulic and *p*-hydroxybenzoic acids. Afterwards, other enzymes induce the forming of coniferyl-, *p*-coumaryl- and sinapyl-alcohols which polymerize to assemble lingin in the secondary CW [47]. CW polymerization appears from production of free radicals which are resulted from the cracking of the covalent linkage between the hydrogen in the alcohol and the phenolic oxygen. Then, these free radicals contribute to the lignin formation and may even assemble linkages to CW sugar polymers [68]. Free radical networks between polysaccharides and lignin monomeric units may form by ester and ether bonds, non-core lignin, whilst free radical polymerization produces condensed lignin [53]. Lignin monomers and hemicellulosic parts in ligno-hemicellulosic bonds have diverse and complex nature which makes difficult to characterize the secondary cell formations for every plant; by reason of this difficulty, studies about this subject are based on grasses (family *Gramineae*). Sun et al. [69] have noted that lignin is strongly bound to polymeric carbohydrates in the CWs of plants by diverse links, such as the covalent bond, which is the alcoholic hydroxyl of the saccharides with primary hydroxyl group at the α-ether linkages of the lignin. Through lignification, the CWs of plants are strengthened by linking of monolignol ferulate, assembling other bonds between structural hemicellulose and lignin [70]. Non-core lignin monomers, like ferulic and *p*-coumaric acids attach core lignin and hemicellulose [71]. Ferulate and 5-5 coupled diferulate copolymerize intensely and form less ether-linked structures with coniferyl alcohol than 8-8′, 8-5′, 8-*O*-4 and 5-5′-coupled diferulates [70,72]. The diminution in ferulate–xylan, ferulate–pectin and ferulate–lignin cross-linking would essentially increase the enzymatic hydrolysis of CWs [73], probably facilitating the liberation of phenols maintained in the CW plant tissue. Other phenolic compounds, such as *p*-coumaric acids are also responsible for ether and ester cross-breed bonds between lignin and saccharides [74], but it still remains not clear how precisely grape phenols are connected and/or tangled in the lignin–polysaccharide network of the grape skin CW.

#### 2.1.2. Phenolic Compounds

In grape skin, phenolic compounds are established in the inner stratum (hypodermis) of the CW and may be classified into [3]:

1. phenolic compounds which are localized in the CW, are bounded to polysaccharides by hydrophobic interactions or hydrogen bonds
2. phenolic compounds which are not localized in the CW, are retained in the vacuoles of plant cells or bounded to the cell nucleus

Table 2 represents the main phenolic compounds present in grape skin (*Vitis vinifera* L.). The concentration of phenols depends on several factors, like geographical origin, climate, ripening time, grape variety and technology of grape processing [2].

##### Cell-Wall Linked Phenolic Compounds

Molecular weight, conformational flexibility, stereochemistry and percentage of galloylation of the phenolic molecule are the main structural and compositional parameters which influence the retention of phenolic compounds. In addition to physical characteristics of the CW, such as porosity, topography and chemical composition may also affect the aggregation between phenols and structural CW polysaccharides [76,77]. The most studies about non-covalent interactions of phenolic substances with CW polysaccharides have been realized using structural components (e.g., polymers with structural rigidity) or strawberry CW obtained by special chemical methods [78]. Two techniques of interaction were established to describe the forming of the complex polysaccharide–phenol systems: (1) hydrogen linkages between the hydroxyl groups of phenolic compounds and the oxygen atoms of the cross-linking ether bonds of mono- and disaccharides appear in the CW polysaccharides. Thus, glucan gels would be accomplish to enclose phenols within their pores [75,79]; (2) hydrophobic interactions appearing as a consequence of the capacity of sugar polymers to enhance secondary formations, e.g., gels, which occur in hydrophobic parts. The constituted hollows may be able to involve phenolic substances, as have been shown to appear between β-cyclodextrin and other phenols, such as flavonoids [76,79].

##### Non-Cell-Wall Phenolic Compounds

Phenolic compounds occurring in plants are not always correlated with the plant CWs; thus, late studies have established that phenols can also be found in the vacuoles of plant cells or bounded to the cell nucleus. (1) Cytoplasmic and vacuolar phenolic compounds: recent studies considering vacuolar phenolic components are concentrated on the research of the color of various flowers and plants and thus, directed on anthocyanins which are stored inside vacuoles. Padayachee et al. [80] discovered higher quantity of acylated than non-acylated anthocyanins in cellular vacuoles. In addition, Agati et al. [81] examined the location and functional significance of flavonoids in plants. They have been noted that flavonoids are localized within different cells in plant environment interactions. Moreover, vacuolar flavonoids can exert their antioxidative function when the physical barrier is broke. Anthocyanins and proanthocyanidins in vacuoles contribute to light screening, photoprotection and pigmentation of plants [82]. (2) Phenolic compounds related with the plant cell nucleus: some research has described the combination of phenols, e.g., flavonoids, with the cell nucleus of various plant tissue. Significant quantity of catechin, epicatechin and proanthocyanidins have been found in *A. Thaliana* nuclei and also, in carrot juice concentrate [80,83]. The possible capacity of phenolic compounds to protect DNA against oxidative stress via radical scavenging activity have been reported by Bouriche et al. [84].

### 2.2. Structure and Composition of Grape Seeds

The grape seeds are reported to contain about 3–6% of the total dry weight of grapes [85,86]. The grape seeds are reported to consist of 11% protein, 35% fiber, 3% minerals, 7% water [87], 7–20% lipids [20,88,89,90] and 7% phenolic compounds [30], tocopherols and β-carotene which are found in grape seed oil. Licev et al. [91] have been reported that grape seeds consist of 25–45% water, 34–36% oil, 4–6% tannins, 2–4% phenolic substances, 4–6% nitrogen compounds, 2–4% minerals, 10–11% cellulose, 8–10% pentosans and 25–28% lignin, but Cotea [92] has been noted that grape seeds includes 28–40% water, 28% cellulose, 0.8–1.2% nitrogen compounds, 4–6% tannins, 10–25% oil and 2–4% mineral substances. The concentration of minority compounds may vary depending on the technological process, environmental and cultivar conditions [89,93].

**Figure 3:**
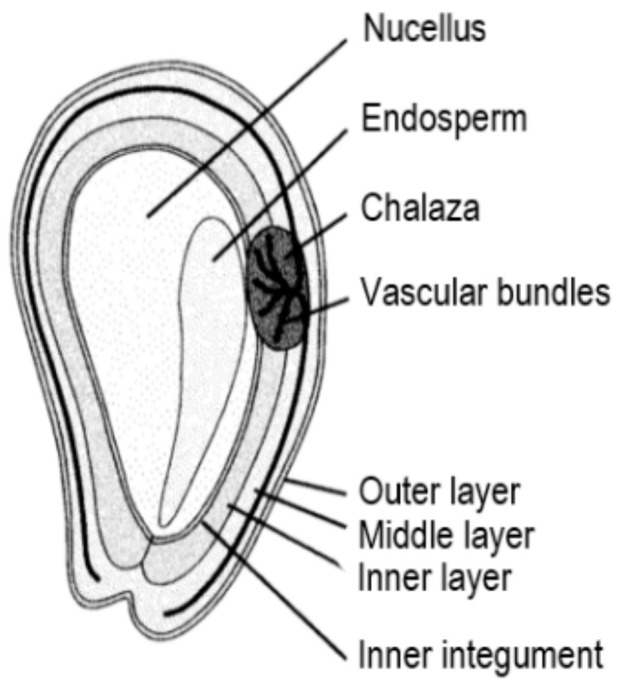
Structure of grape seeds [94].

The structure of grape seeds can be classified into five areas (Figure 3): (1) the cuticle and epidermis; (2) the integument or outer covering of the seed; (3) the middle integument; (4) the inner integument; (5) the endosperm and embryo [94]. Most phenolic compounds are found in the epidermis and outer integument of grape seeds. The grape seeds are a complex matrix which are abundant in valuable compounds, but the most studied components remained grape seed oil and phenols [95].

#### 2.2.1. Grape Seed Oil

Grape seeds contain 8–20% oil [96]. The yield of the grape seed oil depends on the environmental factors and cultivar conditions, grape variety, extraction technique and type of solvent [97,98]. Grape seed oil is abundant in hydrophilic compounds (phenols) and lipophilic compounds (vitamin E, unsaturated fatty acids and phytosterols) [99].

##### Hydrophilic Compounds

Grape seed oil includes phenolic compounds, like flavonoids, carotenoids, phenolic acids, tannins and stilbenes. The total amount of grape seed oil phenols represents 59–360 mg of gallic acid equivalent (GAE)/kg [97,98,100]. The major polyphenols identified in grape seed oil are procyanidin B1, catechin, epicatechin and trans-resveratrol [100]. The quantity of phenolic substances, extracted from grape seed oil by cold-pressing technique, is about 2.9 mg/kg, minor amounts of catechin, epicatechin (1.3 mg/kg each) and trans-resveratrol (0.3 mg/kg) [100]. The low solubility of filtered grape seed oil could be associated to the hydrophilic nature of oil polyphenols. Opposed to this, the unfiltered oil indicated high quantity of polyphenols [100].

##### Lipophilic Compounds

Grape seed oil is composed of mono- and polyunsaturated fatty acids (90%), especially linoleic acid (58–78%), followed by oleic acid (3–15%) [101,102] and minor amounts of saturated fatty acids (10%) [96,103]. Tocotrienols (the unsaturated form of vitamin E) are found in higher amounts in comparison with tocopherols in grape seed oil [104]. Grape seed oil has a high content of vitamin E ranging from 1 to 53 mg/100 g oil [87,105]. The amount of vitamin E depends on the grape variety, environmental and cultivar conditions [87,101]. Other lipophilic compounds which have been found in grape seed oil, are phytosterols. Table 3 shows the main phytosterols in grape seed oil (*Vitis vinifera* L.).

#### 2.2.2. Phenolic Compounds

The concentration of phenolic compounds in grapes is about 2178.8 mg GAE/g (seeds), 374.6 mg GAE/g (skin), 351.6 mg GAE/g (leaves) and 23.8 mg GAE/g (pulp) [107]. The percentage of grape extractable compounds is distributed as follows: 60–70% in seeds, 28–35% in skin and about 10% in pulp [108]. The main phenolic compounds found in grape seeds are flavonoids, especially flavan-3-ols (49.8% catechin, 26% epicatechin and 9.3% epicatechin 3-*O*-gallate monomers) and their polymers [109]. Through catalytic cleavage of the polymer chains, catechin and epicatechin may form polymers which are called proanthocyanidins. The total content of low molecular weight phenolic compounds which are found in grape seeds varies from 55.1 to 964 mg/100 g seeds. Standardized grape seed extracts consist of 74–78% proanthocyanidins and less than 6% of free flavonol monomers related to the dry weight basis, which can subsequently bind to gallic acid and form esters, then glycosides [110,111]. Other phenolic compounds which are presented in grape seeds, are the precursors of phenolic acid (gallic acid) and stilbenes [112]. Rodríguez Montealegre et al. [113] reported that gallic acid in grape seed extracts (*Vitis vinifera* L.) was found in a proportion of 6.8–9.8 mg/kg and protocatechuic acid had values between 3.3 and 8.7 mg/kg. The main stilben identified in grape seeds is trans-resveratrol, having a content less than 0.01 mg/g on a dry weight basis [31]. This variation of phenolic substances is simultaneously influenced by several factors, such as the genetic potential for polyphenol biosynthesis, grape variety, grape berry ripening process, agro-climatic conditions and extraction methods [114,115,116]. In general, grape seeds consisted of lower content of phenolic acids than grape skin, but are more abundant in catechins and procyanidins [19].

### 2.3. Structure and Composition of Grape Inflorescence Architectures

**Figure 4:**
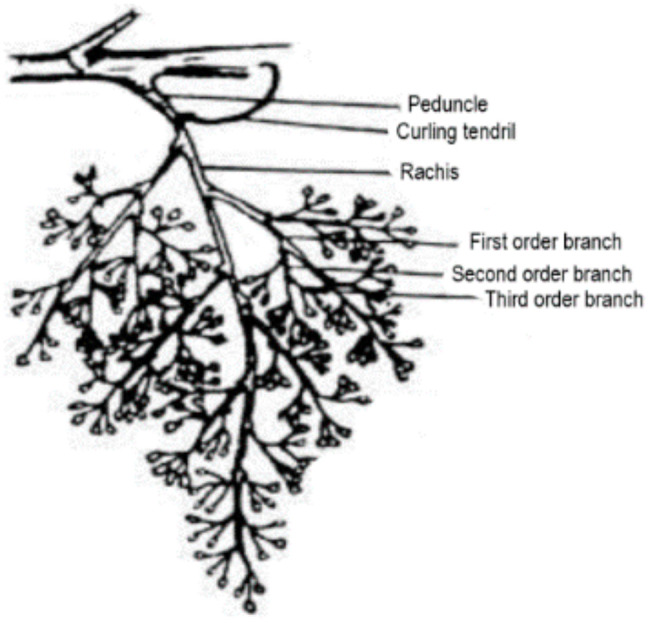
Structure of grape inflorescence architectures [128].

The grape inflorescence architectures are composed of stalk and flowers. The stalk consists of the peduncle, main axis of the inflorescence (rachis) and branches. The main axis of the grape inflorescence contains secondary branches which are extended into tertiary branches with multiple flowers (Figure 4). The architectural variation of grape inflorescences affects fertilization, fruit development, dispersal and crop yield [117,118,119,120,121]. Inflorescence architectures represent about 3–6% of the raw material which are processed in the wine industry [122,123]. These branches contain ligno-cellulosic compounds, such as cellulose, hemicellulose [44], 6–7% tannins [43,124] and 22–47% lignin [42]. According to Prozil et al. [125], the inflorescence branches are composed of 30.3% cellulose, 21% hemicellulose, 17.4% lignin, 15.9% tannins and 6.1% proteins, more than 20 metal cations and monosaccharides. Glucose and xylose are the main sugars found in the inflorescence branches [42,126]. Other monosaccharides, such as mannose, arabinose and galactose were found in minor amounts [44,126]. The concentration of these compounds depends on the geographical origin, climate, harvest time and grape variety [127].

#### 2.3.1. Ligno-Cellulosic Compounds

The main ligno-cellulosic compounds of the inflorescence branches are cellulose, hemicellulose and lignin [6,43]. Cellulose occupies an important part as the most abundant biopolymer, about 30–33% of the total ligno-cellulosic substances [39,125,129], followed by hemicellulose and lignin. The degree of crystallinity of cellulose detected in the inflorescence branches is 75.4% which are higher than in wood (55–65%), but very close to the degree of crystallinity of bacterial and cotton cellulose [129,130]. Access to crystalline cellulose limits the efficiency of enzymatic hydrolysis and microbiological digestion of cellulose in the grape branches [43,131], but due to high degree of crystallinity increases the strength of cellulosic fiber. The second ligno-cellulosic component is xylan which is a group of hemicelluloses. Isolated xylan from the inflorescence architectures contains 89% xylose, 5.5% glucose, 4.9% uronic acid, 0.5% rhamnose and residues of arabinose and galactose [125]. The presence of glucose in isolated xylan can be explained by structural association with xylan or by sorption in solution during ethanol precipitation. The lignin found in the inflorescence branches is of the HGS type which has the following molar ratios 3:71:26 units of *p*-hydroxyphenyl (H), guaiacyl (G) and syringyl (S), respectively. Structural analysis of lignin indicated the predominance of β-*O*-4′ structures (39% mol) and minor amounts of β-5′, β-β, β-1′, 5-5′ and 4-*O*-5′ structures [132].

#### 2.3.2. Phenolic Compounds

In the grape inflorescence architectures, the most abundant phenolic compounds are tannins, which forms mixed polymers with procyanidins (polymers of catechin and epicatechin) and prodelphinidins (polymers of gallocatechin and epigallocatechin) with a high degree of condensation [43,124]. The concentration of tannins depends on their composition and purity. Souquet et al. [133] concluded that tannins in the inflorescence branches have a total concentration of 0.22–0.9 mg/g (approximately 80%), 0.06 mg/g catechin, 0.28 mg/g epicatechin and 0.01 mg/g epigallocatechin. According to Cruz et al. [134] and Makris et al. [124], condensed (non-hydrolysable) and gallic (hydrolysable) tannins were detected in the inflorescence branches.

## 3. Grape Pomace Pectic Substances

Polysaccharides are composed of monosaccharide molecules which are linked in chains by glycosidic bonds and can form linear and branched chains. About 90% of the total number of natural polysaccharides are obtained from plants. Fibers are a category of carbohydrates that cannot be digested and are classified into insoluble (cellulose, hemicellulose, lignin etc.) and soluble (pectin, inulin, gums and mucilage) fibers. Cereals, fruits, vegetables and nuts are natural sources of fiber [135]. The grain, fruit and vegetables residues are the most studied substrates in the food industry regarding to fiber extraction [136]. Dry and wet processing, chemical, physical, gravimetric, enzymatic methods and the combination of these techniques are used for fiber extraction [137], but these methods could change the structure and functionality of the extracted fiber. Nowadays, the new techniques, such as ultrasound, microwave and high voltage electrical discharges which are used for the fiber extraction, reduce the extraction time and increase the content of soluble fiber, especially pectin [138]. Apple pomace and citrus peel are the most traditional materials which are used for pectin extraction. The extracted pectin is slightly different with a multitude of applications in food industry [139]. Dried apple pomace contains about 15–20% pectin, while citrus peel has a range of 30–35% pectin [140]. Unconventional sources of pectin are tropical fruit peels, vegetable residues and different plants [141].

Grape pomace is a rich source of fiber (43–75%), including cellulose, hemicellulose, lignin and pectin. Grape seeds are richer in fiber than grape skin, also red grape pomace has a higher content of fiber in comparison with white variety [142]. Devesa-Rey et al. [5] reported that grape skin is a ligno-cellulosic complex containing a considerable amount of hemicellulose sugars and pectic substances.

### 3.1. Extraction of Pectin from Grape Pomace

The extraction of pectic substances is performed by several methods, such as extraction with solvent, microwave, ultrasound and enzymatic [143,144,145]. Solvents used in pectin extraction are classified into four groups: water and buffers, calcium chelators, acids and bases. Acetic, citric, hydrochloric, nitrogen and sulphuric acids facilitated the extraction of pectin [146,147,148]. The type and concentration of acid affect the yield, physico-chemical and functional properties of pectin [145,149]. Some studies suggested the use of citric acid due to its higher yield and better quality than other acids [150]. Extraction of pectin from guava peel, citrus peel, banana and coffee beans with hydrochloric acid showed the highest yield of pectin compared to other solvents (nitric and citric acid) [151,152,153,154]. Enzymatic extraction of pectin is environmentally safe and more efficient in terms of pectin yield. Different enzymes, such as polygalacturonase, cellulase, xylase, α-amylase etc. are used in pectin extraction [143,155,156]. Enzymes contribute to degradation of pectin and modify the physico-chemical characteristics of pectin. Cellulase has been used to isolate pectin from chicory and cauliflower roots, having a positive effect on the process of cellulose hydrolysis and the release of pectin from the CW [155]. Microwave-assisted extraction has been investigated by many researchers and found that it can promote to a considerable enhancement in the yield and quality of extracted pectin [156,157]. Ultrasonic extraction is an unconventional technique which have a lot of advantages, such as low solvent consumption, shorter extraction time, high pectin extraction yields etc. [158]. A comparative between ultrasound and conventional pectin extraction have showed that ultrasonic treatment increased with 16.32% yield of grapefruit peel pectin in compared to conventional extraction [159]. Du et al. [160] extracted soluble fiber from grape pomace using as solvent, hydrochloric acid and reported that the highest yield (47%) was obtained at 0.4 mol/L of hydrochloric acid, solid to liquid ratio of 1:12, for 90 min and at temperatures below 75 °C. Fereira et al. [161] studied the soluble fibers of Chardonnay grape pomace and observed that extraction with water at high temperatures and solid/liquid ratio of 1:4 facilitated the increasing of pectin content. Minjares-Fuentes et al. [7] performed ultrasonic extraction of pectin from grape pomace using citric acid of pH 2.0 and obtained a yield of pectin (32.3%) at temperature of 75 °C for 60 min. Another study based on the extraction of soluble fiber from grape pomace with hot water was conducted by Beres et al. [21], the optimal extraction conditions being the temperature of 100 °C, solid to liquid ratio of 1:12 and less than 250 nm particle size of grape pomace. Sun et al. [162] studied the soluble fiber of pomace from different grape varieties using extraction with hydrochloric acid and enzymes. Thus, the highest soluble fiber amount was found in Gammay Noir pomace, 455.2 mg/g for hydrochloric acid extraction and 421 mg/g for enzyme extraction, but the lowest content for Syrah variety, 277.2 mg/g and 242.8 mg/g, respectively. Sousa et al. [38] analyzed the composition of grape pomace of the Benitaka variety, obtaining 3.92 g/100 g pectin, a similar content (2.3–4.4 g/100 g) was obtained by Bravo and Saura-Calixto [33]. Another study about the extraction of grape pomace soluble fiber was conducted by Deng et al. [35]. They analyzed the composition of five different varieties of grape pomace (Müller-Thurgau, Morio Muscat, Cabernet Sauvignon, Merlot and Pinot Noir) and obtained 50.6–56.4 mg galacturonic acid equivalent (GUAE)/g for red grape pomace and 32.3–41.2 mg GUAE/g for white grape pomace on a dry weight basis. Colodel et al. [163] analyzed the optimizing conditions for the extraction with nitric acid of pectin from Chardonnay grape pomace. The highest extraction yield (11.1%) was obtained at the correlation between pH 2.0, solid/liquid ratio of 35 mL/g and 135 min. Table 4 presents the soluble fiber/pectin content of different grape pomace, technique and extraction conditions.

### 3.2. Pectin Characterization of Grape Pomace

However, some studies suggested the use of citric acid because of its higher yield and better quality than other acids. Grape pomace represents a major interest in the extraction of soluble fiber, especially pectin, as an alternative source to conventional ones. The characterization of the physico-chemical properties of grape pectin allows the application in food industry, as a hydrocolloid. Bravo and Saura-Calixto [33] analyzed the composition of grape pomace, obtaining a pectin content of 2.3–4.4 g/100 g, 6–9% mineral substances, 12–14% protein and less than 3% soluble carbohydrates. Minjares-Fuentes et al. [7] extracted pectin from grape pomace and obtained 32.3% pectin with a weight of 163.9 kDa, an esterification degree of 55.2% and a concentration of 97% of galacturonic acid units. Another study about composition of grape pomace was conducted by Beres et al. [21]. They reported a content of 3–10% polysaccharides with a composition of the monosaccharides ramnose:xylose:mannose:galactose:glucose:galacturonic acid in a molar ratio of 3:32:2:13:11:20:19. Sousa et al. [38] characterized the chemical composition and bioactive compounds of Benitaka grape pomace (*Vitis vinifera* L.). In the composition of analyzed grape pomace, 3.92 g/100 g of pectin and 9.76 g/100 g of soluble fiber were identified. A detailed analysis of five different grape pomace was performed by Deng et al. [35]. The total pectin content of grape pomace (Müller-Thurgau and Morio Muscat) was 32.3–41.2 mg GUAE/g and for red grape pomace (Cabernet Sauvignon, Pinot Noir and Merlot) a content of 50.6–56.4 mg GUAE/g on a dry weight basis. Iora et al. (2015) analyzed the physico-chemical properties of grape pomace, obtaining 6.99%, 4.59% and 3.46% for Merlot, Tanat and Cabernet varieties, respectively. The most recent study based on the comparative evaluation of pectin substances in grape pomace was conducted by Limareva et al. [170]. The total pectin content detected in grape pomace was 3.21–7.27%. The lowest yield of hydropectin (0.9%) was achieved in Cabernet Sauvignon grape pomace and the highest (2.01%) in Saperavi Severnyi grape pomace. Grape pomaces of Saperavi Severnyi and Moldova presented an amount of 69.09% and 59.93% polygalacturonic acid, respectively. Other grape varieties presented an average of 42.8% polygalacturonic acid. Experimental data showed values between 3.7 and 6.8% of methoxyl compounds. These values describe a low gelling capacity of pectin. Acetyl compounds presented a range of values from 0.1% (Saperavi Severnyi) to 0.39% (Moldova). A high content of free carboxyl groups (1–4%) was found in the extracted pectin, which may indicate a great capacity of pectin complexation. The degree of esterification ranged from 52% to 65%. Thus, the complexing properties of pectin substances presented values from 75 (Cabernet Sauvignon and Moldova) to 110 mg Pb^2+^/g pectin (Saperavi Severnyi, Chardonnay and Rkatsiteli).

Colodel et al. [163] studied the optimal conditions for extraction of pectin from Chardonnay grape pomace. Extracted pectin from grape pomace had a content of 11.1% and 56.8% uronic acid. The fractioned pectin was consisted of 7.8% rhamnose, 6% arabinose, 13.6% galactose and a minor amount of other neutral monosaccharides. The extracted pectin had a molar mass of 154,100 g/mol with an esterification degree of 18.1%, including 55.7% homogalacturonan and 35.2% rhamnogalacturonan I. Table 5 shows the physico-chemical properties of grape pomace pectin.

## 4. Conclusions

Grape pomace remains one of the main by-product of wine industry rich in ligno-cellulosic compounds, neutral polysaccharides, structural proteins, phenolic and pectic substances. The present systematic review showed the structure and composition of the grape pomace components (skin, seeds and inflorescence architectures) and the potential of grape pomace as an unconventional source of pectin. The utilization of grape pomace as a source of pectic substances is a promising field. In this way, the recovery of pectin, bioactive compounds and oil from grape pomace can still be an attractive field of waste generation and environmental approach. Moreover, grape pomace valorization is a concept increasingly consolidated in the field of recent applications related to the food industry. The potential of the pectin extraction described are compelling reasons for further studies on this topic. The grape pomace can be considered an important source of pectin after citrus and apple pomaces with high application to food industry (e.g., additive for texture/rheology, ingredient for edible films), chemical industry and pharmaceutical industry.

## Figures and Tables

**Table 1:** Physico-chemical composition of grape pomace (*Vitis vinifera* L.).

Compound	Dry Matter Content	References
Physico-chemical parameters		[37,38,39]
Ash	4.65 ± 0.05 g/100 g	
Moisture content	3.33 ± 0.04 g/100 g	
Fiber	46.17 ± 0.80 g/100 g	
Lipids	8.16 ± 0.01 g/100 g	
Proteins	8.49 ± 0.02 g/100 g	
Carbohydrates	29.20 g/100 g	
Fructose	8.91 ± 0.08 g/100 g	
Glucose	7.95 ± 0.07 g/100 g	
Energy value	224.00 Kcal/100 g	
Mineral substances		
Ca	9.90 g/kg	
P	2.70 g/kg	
Mg	0.80 g/kg	
K	13.90 g/kg	
Na	0.22 g/kg	
S	1.50 g/kg	
Mn	13.00 mg/kg	
Zn	25.00 mg/kg	
Cu	49.00 mg/kg	
Fe	361.00 mg/kg	
Se	0.20 mg/kg	
Co	0.40 mg/kg	
Bioactive compounds		
Vitamin E	5.00 mg/kg	
Vitamin C	26.25 ± 0.01 mg AAE ^a^/g	
Soluble fiber	9.76 ± 0.03 g/100 g	
Insoluble fiber	36.40 ± 0.84 g/100 g	
Total anthocyanin content	131.00 ± 0.40 mg/100 g	
Total phenolic content	60.10 ± 0.10 mg GAE ^b^/g	
Catehic tannins	13.10 ± 0.80 mg CE ^c^/g	
Hydrolysable tannins	3.70 ± 0.10 mg TAE ^d^/g	
Quercitin	128.70 ± 5.90 μg/g	
Gallic acid	607.00 ± 9.00 μg/g	
Catechin	1973.40 ± 17.60 μg/g	
Procyanidin B2	1071.00 ± 17.70 μg/g	

^a^ AAE—ascorbic acid equivalent; ^b^ GAE—gallic acid equivalent; ^c^ CE—catechin equivalent; ^d^ TAE—tannic acid equivalent.

**Table 2:** Main phenolic compounds occurring in grape skin (*Vitis vinifera* L.).

Phenolic Compounds		Content, mg/g	References
Free phenolic compounds	Gallic acid	13.7 ± 0.6	[3,75]
	Caftaric acid	40.4 ± 3.6	
	Protocatechuic acid	11.0 ± 1.0	
	Vanillic acid	9.2 ± 2.4	
	Caffeic acid	n.d.	
	Syringic acid	4.3 ± 0.1	
	*p*-Coumaric acid	n.d.	
	(+)-Catechin	16.5 ± 0.6	
	(−)-Epicatechin	23.7 ± 1.4	
	Rutin	143.1 ± 7.6	
	Isoquercitrin	212.1 ± 12.8	
	Kaempferol	362.7 ± 45.0	
	Resveratrol	149.2 ± 11.0	
Bound phenolic compounds	Gallic acid	2.6 ± 0.1	
	Caftaric acid	7.4 ± 0.1	
	Protocatechuic acid	4.4 ± 0.2	
	Vanillic acid	4.9 ± 0.6	
	Caffeic acid	7.2 ± 0.3	
	Syringic acid	2.2 ± 0.1	
	*p*-Coumaric acid	6.4 ± 1.9	
	(+)-Catechin	13.6 ± 1.0	
	(−)-Epicatechin	10.6 ± 1.9	
	Rutin	4.5 ± 1.2	
	Isoquercitrin	3.4 ± 1.1	
	Kaempferol	46.4 ± 0.9	
	Resveratrol	0.3 ± 0.1	

n.d.—non-detected.

**Table 3:** Main phytosterols in grape seed oil (*Vitis vinifera* L.).

Phytosterols	Content, mg/kg Oil	References
Cholesterol	n.d.-10.00	[87,106]
Cholestanol	n.d.	
Brassicasterol	0.60–0.90	
2,4-methylene-cholesterol	n.d.-0.18	
Campesterol	0.10–9.30	
Campestanol	n.d.	
Stigmasterol	10.20–10.80	
Δ-7 campesterol	0.16–0.27	
Δ-5 2,3-stigmastadienol	n.d.	
Clerosterol	0.90–0.94	
β-sitosterol	66.60–67.40	
Sitostanol	3.92–4.70	
Δ-5 avenasterol	1.98–2.09	
Δ-5 2,4-stigmastadienol	0.41–0.47	
Δ-7 estigmastenol	1.99–2.30	
Δ-7 avenasterol	0.98–1.10	

n.d.—non-detected.

**Table 4:** Grape pomace soluble fiber/pectin content and extraction techniques.

Grape Pomace Variety	Yield of Soluble Fiber/Pectin Recovery		Extraction Technique and Operating Conditions	References
*Vitis vinifera* L.	2.3–4.4 g/100 g pectin		n.d.	[33]
Manto Negro	6.20 ± 0.30% soluble pectins		Solvent extraction Solvent: 0.5 M hydrochloric acid; temperature: 80 °C	[164]
Prensal Blanc	45.0 ± 1.6 g/kg soluble pectins		Solvent extraction Solvent: 0.5 M hydrochloric acid; temperature: 80 °C	[165]
Gamay Noir	Soluble dietary fiber	455.2 mg/g	Solvent extraction Solvent: hydrochloric acid; solid/liquid ratio: 1:20; temperature: 75 °C; time: 75 min	[162]
Chardonnay		438.6 mg/g		
Syrah		277.2 mg/g		
Gamay Noir		421.0 mg/g	Enzyme-assisted extraction Enzyme: cellulase; solid/liquid ratio: 1:20; temperature: 55 °C; time: 210 min	
Chardonnay		401.8 mg/g		
Syrah		242.8 mg/g		
*Vitis vinifera* L.	37–54 mol% pectic substances of CWP ^a^		Solvent extraction Solvent: sulphuric acid; temperature: 20 °C; time: 3 h	[4]
*Vitis vinifera* L.	47% soluble fiber		Solvent extraction Solvent: hydrochloric acid; solid/liquid ratio: 1:12; temperature: 75 °C; time: 90 min	[160]
RWGP ^b^ (Cabernet Sauvignon, Merlot, Pinot Noir)	32.3–41.2 mg GUAE ^d^/g total extractable pectins		Solvent extraction Solvent: deionized water; solid/liquid ratio: 1:20; time: 10 min	[35]
WWGP ^c^ (Muller Thurgau, Morio Muscat)	50.6–56.4 mg GUAE/g total extractable pectins			
Pinot Noir Merlot	3.68 ± 0.05% pectin 5.82 ± 0.81% pectin		Solvent extraction Solvent: water; time: 10 min	[166]
Chardonnay	0.904 ± 0.045 g/100 g mass of sugar		Solvent extraction Solvent: water; solid/liquid ratio: 1:4; temperature: 90 °C; time: 3 h	[161]
	2.156 ± 0.012 g/100 g mass of sugar		Solvent extraction Solvent: 2% alkali solution; solid/liquid ratio: 1:4; temperature: 90 °C; time: 5 h	
	0.757 ± 0.010 g/100 g mass of sugar		Enzyme-assisted extraction Enzyme: cellulase; solid/liquid ratio: 1:4; temperature: 50 °C; time: 3 h	
Cabernet Sauvignon	32.4 ± 1.4% pectin		Ultrasound-assisted extraction Solvent: citric acid; pH 2.0; solid/liquid ratio: 1:10; temperature: 75 °C; time: 60 min; frequency: 37 kHz	[7]
Benitaka	3.92 ± 0.02 g calcium pectate/100 g		Neutralization the overall charge of free uronic acid residues by calcium ions	[38]
Merlot	6.99 ± 0.19% pectin		Solvent extraction Solvent: phosphate-citrate buffer; pH 3.0; solid/liquid ratio: 1:50; temperature: 80 °C; time: 2 h	[167]
Tanat	4.59 ± 0.18% pectin			
Cabernet	3.46 ± 0.21% pectin			
Pinot Noir	10.93% total sugar		Solvent extraction Solvent: water; solid/liquid ratio: 1:12; temperature: 100 °C; time: 1 h; particle size: <249 μm	[21]
*Vitis vinifera* L.	11 g CASS ^e^/g pectic polysaccharides 8 mg DASS ^f^/g pectic polysaccharides		Solvent extraction Solvent (CASS): 50 mM trans-1,2-diaminocyclohexane-N,N,N,N-tetraacetic acid; time: 6 and 12 h Solvent (DASS): 50 mM sodium carbonate/20 mM sodium borohydride; temperature: 4 °C; time: 6 and 12 h	[168]
Cabernet	11.25 g/100 g soluble dietary fiber		n.d.	[169]
Chardonnay	11.1% pectin		Solvent extraction Solvent: nitric acid; pH: 2.08; solid/liquid ratio: 35.11 mL/g; time: 135.23 min	[163]
Cabernet Sauvignon	1.9% protopectin 0.9% hydropectin		Calcium-pectatism method	[170]
Saperavi Severnyi	2.85% protopectin 2.01% hydropectin			
Moldova	3.95% protopectin 1.8% hydropectin			
Aligote	2.4 protopectin 1.23% hydropectin			
Chardonnay	2.3 protopectin 1.3% hydropectin			
Rkatsiteli	2.2% protopectin 1.2% hydropectin			
Pervenets Magarachea	2.6 protepctin 1.1% hydropectin			

n.d.—non-determined; ^a^ CWP—cell wall polysaccharides; ^b^ RWGP—red wine grape pomace; ^c^ WWGP—white wine grape pomace; ^d^ GUAE—galacturonic acid equivalent; ^e^ CASS—chelating agent soluble solids; ^f^ DASS—dilute alkaline soluble solids.

**Table 5:** Physico-chemical properties of grape pomace pectin.

Grape Pomace Variety	Galacturonic Acid Content	Degree of Esterification	Molecular Weight	Neutral Monosaccharide Content	References
Manto Negro	6.21 ± 0.18%	n.d.	n.d.	4.60 ± 0.12%	[164]
Prensal Blanc	41.8 ± 1.6 g/kg (uronic acids)	n.d.	n.d.	61.2 ± 1.7 g/kg	[165]
Cabernet Sauvignon	n.d.	21–39%	n.d.	0.2 Rha ^a^, 0.4 Fuc ^b^, 5.1 Ara ^c^, 11.6 Xyl ^d^, 7.4 Man ^e^, 3.6 Gal ^f^, 40.7 Glc ^g^ (mol%)	[4]
Callet				0.2 Rha, 1.0 Fuc, 4.9 Ara, 17.2 Xyl, 6.2 Man, 3.3 Gal, 38.3 Glc (mol%)	
Manto Negro				0.3 Rha, 0.3 Fuc, 6.9 Ara, 11.5 Xyl, 5.6 Man, 4.9 Gal, 38.5 Glc (mol%)	
Merlot				0.1 Rha, 0.3 Fuc, 5.5 Ara, 19.2 Xyl, 7.4 Man, 3.3 Gal, 34.6 Glc (mol%)	
Tempranillo				0.2 Rha, 0.3 Fuc, 4.8 Ara, 12.8 Xyl, 4.6 Man, 3.5 Gal, 38.4 Glc (mol%)	
Syrah				0.2 Rha, 0.1 Fuc, 4.8 Ara, 18.4 Xyl, 6.0 Man, 3.5 Gal, 37.7 Glc (mol%)	
Chardonnay				0.1 Rha, 0.1 Fuc, 6.4 Ara, 14.1 Xyl, 4.8 Man, 3.9 Gal, 29.8 Glc (mol%)	
Macabeu				0.1 Rha, 0.1 Fuc, 6.0 Ara, 8.4 Xyl, 5.6 Man, 4.0 Gal, 35.9 Glc (mol%)	
Parellada				0.2 Rha, 0.2 Fuc, 6.9 Ara, 12.0 Xyl, 5.7 Man, 4.0 Gal, 38.7 Glc (mol%)	
Manto Negro	n.d.	21–39%	n.d.	0.1 Rha, 0.1 Fuc, 6.2 Ara, 11.5 Xyl, 4.7 Man, 3.9 Gal, 35.6 Glc (mol%)	[4]
Muller Thurgau	0.43 ± 0.06 mg GUAE ^h^/g	n.d.	n.d.	0.07% Ara, 0.02% Xyl, 0.05% Man, 0.12% Gal, 0.20% Glc	[35]
Morio Muscat	0.26 ± 0.03 mg GUAE/g			0.11% Ara, 0.02% Xyl, 0.01% Man, 0.13% Gal, 0.15% Glc	
Cabernet Sauvignon	0.27 ± 0.04 mg GUAE/g			0.07% Ara, 0.03% Xyl, 0.05% Man, 0.07% Gal, 0.33% Glc	
Merlot	0.73 ± 0.07 mg GUAE/g			0.13% Ara, 0.04% Xyl, 0.09% Man, 0.15% Gal, 0.38% Glc	
Pinot Noir	0.72 ± 0.06 mg GUAE/g			0.16% Ara, 0.02% Xyl, 0.22% Man, 0.18% Gal, 0.43% Glc	
Merlot	0.50 ± 0.07% (uronic acid)	n.d.	n.d.	0.73 ± 0.01% (total content of neutral sugar)	[166]
Pinot Noir	0.35 ± 0.04% (uronic acid)			1.09 ± 0.01% (total content of neutral sugar)	
Chardonnay	n.d.	n.d.	n.d.	4.6–8.7 Rha, 3.1 Fuc, 22.1–30.9 Ara, 3.5–7.6 Xyl, 7.6–9.8 Man, 8.6–19.7 Gal, 20.2–51.4 Glc (mol%)	[161]
Cabernet Sauvignon	n.d.	55.2%	163.9 kDa	n.d.	[7]
Pinot Noir	31 mol% (GalA ^i^)	n.d.	n.d.	2.0–40.5 Rha, 20.4–38.4 Ara, 1.2–3.3 Xyl, 10.3–14.9 Man, 6.6–28.3 Gal, 6.0–37.0 Glc (mol%)	[21]
Chardonnay	56.8 ± 0.3% (GalA)	43.3%	1.597 × 10^5^ g/mol	2.7% Rha, 0.2% Fuc, 2.9% Ara, 2.5% Xyl, 2.2% Man, 7.2% Gal, 25.5% Glc	[163]
Saperavi Severnyi	42.8–69.09%	52–65%	n.d.	n.d.	[170]
Moldova					
Cabernet Sauvignon					
Aligote					
Chardonnay					
Rkatsiteli					
Pervenets Magarachea					

n.d.—non-determined; ^a^ Rha—rhamnose; ^b^ Fuc—fucose; ^c^ Ara—arabinose; ^d^ Xyl—xylose; ^e^ Man—mannose; ^f^ Gal—galactose; ^g^ Glc—glucose; ^h^ GUAE—galacturonic acid equivalent; ^i^ GalA—galacturonic acid.

## Data Availability

Not applicable.
